# Emotional Eating, Impulsivity, and Affective Temperaments in a Sample of Obese Candidates for Bariatric Surgery: Which Linkage?

**DOI:** 10.3390/brainsci15040372

**Published:** 2025-04-03

**Authors:** Davide Gravina, Miriam Violi, Andrea Bordacchini, Elisa Diadema, Sara Fantasia, Marly Simoncini, Claudia Carmassi

**Affiliations:** 1 Department of Clinical and Experimental Medicine, University of Pisa, 56127 Pisa, Italy; miriamvioli@gmail.com (M.V.); andreabordacchini@gmail.com (A.B.); e.diadema@studenti.unipi.it (E.D.); dr.fantasiasara@gmail.com (S.F.); marlysimoncini@virgilio.it (M.S.); claudia.carmassi@unipi.it (C.C.); 2 Department of Biotechnology Chemistry and Pharmacy, University of Siena, 53100 Siena, Italy

**Keywords:** obesity, bariatric surgery, emotional eating, disordered eating behaviors, problematic eating behaviors, impulsivity, affective temperaments

## Abstract

**Background/Objectives**: Obesity is a major public health challenge of the 21st century, with prevalence rates steadily rising globally. Disordered eating behaviors, particularly emotional eating (EE), complicate the clinical management of obesity and hinder long-term outcomes, such as maintaining weight loss after bariatric surgery. Studies reveal that EE affects 65–75% of overweight or obese adults, and such behavior may stem from a disrupted brain reward system linked to emotional dysregulation and impulsivity. Impulsivity in obesity involves deficient cognitive inhibitory control, creating an imbalance between impulsive and reflective systems. While problematic eating behaviors and obesity are well studied, the role of affective temperaments—innate traits influencing mood, energy, and responses to stimuli—remains underexplored. This study aims to examine the interplay between emotional eating, impulsivity, and affective temperaments in obese patients preparing for bariatric surgery. **Methods**: A total sample of 304 obese outpatients was consecutively enrolled at the Psychiatry Clinic of the Department of Clinical and Experimental Medicine of the University of Pisa during the presurgical mental health evaluation routinely performed before the bariatric intervention. Sociodemographic and clinical data were collected by psychiatrists during a single consultation. Assessments also included the following psychometric tests: the Structured Clinical Interview (SCID-5), the Emotional Eating Scale (EES), the Barratt Impulsivity Scale-Version 11 (BIS-11), and the Temperament Evaluation of Memphis, Pisa, Paris, and San Diego-Auto-questionnaire (TEMPS-A). **Results**: A significant correlation was observed between the EES total score and the BIS total score (*p* = 0.003), as well as with the sub-dimensions of attentional impulsivity (*p* < 0.001) and motor impulsivity (*p* = 0.024). In addition, a significant correlation has been found between the total score of EES and the cyclothymic (*p* < 0.001), depressive (*p* < 0.001), irritable (*p* = 0.013), and anxious (0.020) temperaments. When comparing obese patients with EE and without EE (No-EE), higher rates of both current (*p* = 0.007) and lifetime (*p* = 0.024) psychiatric comorbidities were observed in the EE group, namely for anxiety disorders (*p* = 0.008) and eating disorders (*p* = 0.014). **Conclusions**: Our study highlights a significant association between EE in obese patients with the cyclothymic, irritable, anxious, and depressive temperaments, and impulsivity dimension. Thus, problematic eating behaviors and temperamental traits may have a bidirectional psychopathological influence in obese patients and need to be carefully evaluated in subjects seeking bariatric surgery.

## 1. Introduction

Obesity represents one of the main concerns for both clinical and public health spheres globally and has become one of the major public health challenges of the 21st century [1]. Indeed, its prevalence has surged dramatically, reaching epidemic proportions [2,3,4]. The World Health Organization (WHO) reported in 2016 that over 1.9 billion adults were overweight, with more than 650 million classified as obese, and these numbers have been growing steadily over the last years. The pathogenesis of this disorder is complex and multifactorial, including genetic, biological, metabolic, environmental, and psychological factors [5]. Within the context of obesity, disordered eating behaviors (DEBs) turned out to play a pivotal role, further complicating the clinical management of this condition and representing a maintaining factor of the disorder [6,7]. Among these behaviors, emotional eating (EE) has garnered particular attention as a stronger predictor of binge-eating disorder (BED) and obesity [8,9]. EE refers to the tendency to consume food in response to negative emotions rather than hunger cues. This behavior often involves high-calorie, palatable foods that provide immediate but temporary relief from emotional distress. Chronic stress plays a significant role in driving EE by altering hormonal pathways, including the hypothalamic-pituitary-adrenal (HPA) axis. Elevated cortisol levels, a hallmark of stress, increase appetite and cravings for high-fat and high-sugar foods [10,11]. Furthermore, EE can create a vicious cycle: negative emotions trigger overeating, leading to guilt or shame, which then reinforces the behavior. Indeed, EE has been linked to the onset of “food addiction” in susceptible individuals, which could escalate into severe forms of binge eating characterized by multiple episodes of overeating, disinhibition, psychological distress, impulsivity, and emotional dysregulation [12]. In fact, evidence from the literature highlights how pathological overeating may be a manifestation of a disrupted brain reward system, similarly to the biological pathways involved in substance use disorders (SUDs). This has been related to phenotypic expressions characterized by high emotional dysregulation and impulsivity [13]. The impulsiveness dimension observed in eating disorders (EDs) and obesity has been accounted for by a deficient top–down cognitive inhibitory control over salience stimuli, with a subsequent imbalance between impulsive and reflective systems [14,15]. Indeed, as many as 54% of patients seeking bariatric surgery exhibit symptoms indicative of food dependence [16]. This represents an obstacle for effective interventions, especially for long-term weight maintenance. For this reason, research has largely investigated the role of problematic eating behaviors as possible predictors of good outcomes for bariatric surgery in people with severe obesity [17,18,19,20], and their influence on weight loss and weight loss maintenance after surgery [21]. Similarly, the strong association between obesity and mental disorders has long been investigated [22]. Studies indicate that 37% to 81% of obese patients seeking bariatric surgery have experienced at least one psychiatric disorder during their lifetime [23], with EDs, mood disorders (MDs), and anxiety disorders being the most prevalent [23,24,25,26,27].

However, even in obese patients eligible for bariatric surgery who do not show overt psychiatric disorders, there may be sub-threshold and unrecognized psychopathological elements that could play a decisive role in the trajectory of the disorder. In this regard, the role of affective temperaments has not yet been thoroughly investigated and results from the literature are not always consistent. Affective temperaments are innate emotional predispositions characterized by relatively stable, stereotypical patterns of emotion-related traits that serve evolutionary functions [28]. These temperaments are thought to arise from a genetic or structural foundation, influencing energy levels, mood, and how individuals respond to external stimuli [29]. In contrast, temperament differs from character, which is shaped by cultural influences and life experiences. Together, temperament and character form the components of personality. According to Akiskal et al. [30], five affective temperaments have been identified: hyperthymic, cyclothymic, dysthymic, irritable, and anxious.

While temperaments themselves do not constitute disorders, pronounced temperamental traits may predispose individuals to mood episodes and can influence their specific clinical presentation [31]. The relationship between obesity and affective temperaments has been slightly investigated, but there is currently still a dearth in scientific literature of solid evidence on this topic. A cross-sectional study [32] highlighted how binge-eating episodes are more frequently associated with cyclothymic, dysthymic, and hyperthymic temperamental dimensions. Likewise, another cross-sectional study from 2009 revealed elevated levels of cyclothymic, anxious, and irritable temperamental traits in bariatric patients [33]. However, more recent research identified an inverse relationship between body mass index (BMI) and cyclothymic temperament, along with a direct positive association between BMI and hyperthymic temperament [34].

A better understanding of the psychopathological dimensions determining altered eating behavior in patients with obesity could provide a better clinical characterization of the disorder and potentially have important implications regarding outcomes and the possibility of delivering targeted interventions. Considering the above, the aim of this study is to investigate the relationship between emotional eating, impulsivity, and affective temperaments in a sample of obese patients candidates for bariatric surgery.

## 2. Materials and Methods

### 2.1. Sample Recruitment

A total sample of 304 obese outpatients who underwent clinical evaluation for bariatric surgery (Roux-en-Y Gastric Bypass, RYGBP, or Gastrectomy Sleeve) was recruited at the University Hospital of Pisa. Enrollment exclusion criteria were: (a) age under 18 or over 75 years old; (b) mood disorder induced by medical or neurological conditions; (c) difficulty in understanding the questionnaires proposed due to language barriers or intellectual deficits; (d) impossibility of providing informed consent.

According to international guidelines from the National Institutes of Health consensus statement [35,36,37], the pre-operative multidisciplinary assessment should include a mental health evaluation to investigate any psychiatric disorders or maladaptive psychosocial factors that could contraindicate surgery. Thus, participants were consecutively evaluated at the Psychiatry Section of the Department of Clinical and Experimental Medicine of the University of Pisa. Key demographic information was collected for each subject, including gender, age, marital status, level of education, and occupation. An accurate psychiatric evaluation was carried out by a specialist in psychiatry, collecting clinical psychiatric history with particular attention to current and lifetime psychiatric disorders, obesity-related clinical and physical features, BMI, weight trend, and eating behaviors over time. Written informed consent was obtained from all eligible participants after they were provided with a detailed explanation of the study and given the opportunity to ask questions. Therefore, a battery of psychometric tests was also administered to enrolled patients to collect data for the study and complete the psychopathological framework. The study was conducted in accordance with the Declaration of Helsinki, and the Ethics Committee of the Azienda Ospedaliero-Universitaria of Pisa approved all recruitment and assessment procedures.

### 2.2. Assessment Instruments

#### 2.2.1. Sociodemographic and Clinical Data

A Case Report Form (CRF) was specifically designed to collect participants’ sociodemographic data (including age, gender, marital status, education, and employment) and clinical variables (such as BMI, previous psychiatric diagnoses, and psychopharmacological treatments). This data was systematically collected using a custom schedule. The Structured Clinical Interview for the Disorders of DSM-5 (SCID-5 CV) was utilized to confirm psychiatric diagnoses of recruited patients according to DSM-5 criteria [38].

#### 2.2.2. Emotional Eating

Patients were administered the Emotional Eating Scale (EES) to assess the tendency to overfeed in response to emotional states [39]. The survey consists of a 25-item scale measuring overeating in the presence of negative emotional states related to anger, anxiety, and depression. Participants rate the extent to which certain feelings lead to the urge to eat using a 5-point Likert scale ranging from “no desire to eat” to “an overwhelming urge to eat”. According to Arnow’s article [39], participants were classified as high in EES if their score was ≥25, as an EES scale score greater than or equal to 25 indicates that “reliance on food to manage emotions is probably affecting the patient’s quality of life”. The EES showed good reliability and validity, and coefficient alphas for the validation study of 0.89 and 0.85 for the anger and anxiety subscales, respectively [39].

#### 2.2.3. Impulsivity

All participants completed the Barratt Impulsiveness Scale (BIS-11) to evaluate the dimension of impulsivity as a behavioral or personality variable by calculating a total score as an expression of general impulsiveness, based on the sum of first- or second-order factors according to the revised version of the scale [40]. The BIS-11 consists of 30 items, which participants rate on a 4-point Likert scale: Rarely/Never (=1), Occasionally (=2), Often (=3), and Almost Always/Always (=4). The BIS-11 subscales evaluate attentional impulsiveness (inability to focus attention), motor impulsiveness (acting without thinking), and non-planning impulsiveness (lack of orientation to the future). The scale showed good psychometric properties, with high internal consistency [40], and is a widely used and well-validated self-report instrument to measure impulsiveness in selected patient populations.

#### 2.2.4. Affective Temperaments

Affective temperaments were assessed using the 39-item version of the validated Italian Temperament Evaluation of Memphis, Pisa, Paris, and San Diego—Auto-questionnaire (TEMPS-A) [41]. This scale represents the Italian short version of the original TEMPS-A scale, which comprised a total of 110 items, with each of the five temperament dimensions (cyclothymic, depressive, irritable, hyperthymic, and anxious) represented by about 20 items each [42]. This shortened version of the scale consists of 39 items and represents a useful and efficient tool for both research and clinical purposes to assess the five dimensions of temperament, especially when a concise assessment is required. According to Elias et al. [43], good psychometric properties have been highlighted for this assessment instrument. The test–retest reliability of the TEMPS-A ranged from 0.58 for the irritable to 0.68 for the cyclothymic, 0.69 for the dysthymic, and 0.70 for the hyperthymic temperament in the validation study [42]. The instrument showed excellent internal consistency, with Cronbach’s α ranging from 0.76 for the dysthymic to 0.88 for the cyclothymic temperament in the same study. For the Italian short version of the TEMPS-A, Cronbach’s α was 0.79 for the cyclothymic, 0.72 for the depressive, 0.72 for the irritable, 0.75 for the hyperthymic, and 0.71 for the anxious temperament [41].

### 2.3. Statistical Analyses

Descriptive statistics were used to summarize sample characteristics, reported as means and standard deviations (SDs) for continuous variables and as numbers and percentages for categorial variables. The linear correlation between the scale scores was measured with Pearson’s correlation coefficient. Comparisons between groups were made using the Chi-squared test for categorical variables, Student’s *t*-test for continuous variables with a normal distribution, and the Mann-Whitney test for continuous variables without a normal distribution. Statistical significance was set at *p* < 0.05 (two-tailed). We used the statistical routines of IBM SPSS Statistics for Mac, version 25.0 (SPSS Inc., Chicago, IL, USA).

## 3. Results

### 3.1. Socio-Demographic Characteristics

In the overall sample, participants were predominantly female (71.4%), and the mean age was 47 years. Regarding marital status, 14.8% of patients were single at the time of evaluation, while 68.7% were married, 13.2% were divorced, and 3.3% were widowed. Most of the patients had a secondary/elementary school diploma (47.4%) or a high school diploma (41.1%), while only 11.5% of participants were graduated. As far as work status was concerned, most of the patients were employed (79.3%). Finally, the mean BMI of the whole sample was 43.3 kg/m^2^. Sociodemographic characteristics of the sample are summarized in Table 1.

### 3.2. Correlation of Emotional Eating with Impulsivity and Affective Temperaments

To assess the association of emotional eating with clinical features, a linear correlation between the scale scores has been outlined. When investigating the correlation between emotional eating and impulsivity dimensions on the overall sample, a significant correlation was highlighted between the total score of the EES scale with the total score of the BIS scale on impulsivity (*p* = 0.003), along with a significant correlation with two BIS sub-dimensions, namely attentional impulsivity (*p* < 0.001) and motor impulsivity (*p* = 0.024).

Regarding affective temperaments assessed through the TEMPS-A questionnaire, we found a significant correlation between the total score of EES and the cyclothymic (*p* < 0.001), depressive (*p* < 0.001), irritable (*p* = 0.013), and anxious (0.020) temperaments in the entire sample. An overview of outcomes is reported in Table 2. Gender differences for each correlation are provided in Supplementary Materials (Tables S1 and S2).

### 3.3. Comparison Between Obese Patients with (EE) and Without (No-EE) Emotional Eating

To explore the differences in clinical variables between obese patients with and without EE, the total sample was divided into two groups based on the total score recorded on the EES. Therefore, from the whole survey of patients who fulfilled the EES (N = 262), a group of patients with emotional eating (EE, N = 103) and a group of patients without emotional eating (No-EE, N = 159) were identified (Table 3).

No significant differences have been detected regarding sociodemographic variables for the EE group compared to the No-EE group. Conversely, statistically significant findings between the two groups were observed regarding lifetime psychiatric comorbidities and psychiatric family history. In fact, results highlighted significantly higher rates of family history for anxiety disorders (*p* < 0.001), suicidality (*p* = 0.043), and, more generally, for psychiatric disorders (*p* = 0.036) in the EE group.

Regarding psychiatric comorbidities, significantly higher rates of anxiety disorders (*p* = 0.008) were also found in the EE-group. More precisely, significant differences were observed for generalized anxiety disorder (*p* = 0.012) and panic disorder (*p* = 0.049), instead of for social anxiety disorder (*p* = 0.420).

As expected, a significantly higher occurrence of eating disorders was also observed in the EE group compared to No-EE group (*p* = 0.014). However, significance in rate differences for binge-eating disorders (N-EE: 5/159, 3.1%; EE: 8/103, 7.8%; *p* = 0.092) and bulimia nervosa (N-EE: 2/159, 1.3%; EE: 3/103, 2.9%; *p* = 0.339) was not reached between the two groups.

Overall, comorbidities with both current (*p* = 0.007) and lifetime (*p* = 0.024) psychiatric disorders were found to be significantly more represented in the EE –group. Consequently, mood stabilizers (*p* = 0.011) and antidepressants (*p* = 0.036) were also found to be significantly more prescribed in these patients than in the No-EE group.

### 3.4. Emotional Eating, Impulsivity, and Affective Temperaments Between Patients with and Without Severe Obesity (Class III)

In accordance with the international classification of obesity [44], the whole sample was divided based on the severity of obesity. Two groups were thus formed: one consisting of patients with severe obesity (BMI ≥ 40 kg/m^2^, Class III Obesity) and one with patients without severe obesity (BMI < 40 kg/m^2^). The sample distribution of participants who completed the psychometric scales for each group was as follows: 76 patients with BMI < 40 kg/m^2^ and 183 patients with BMI ≥ 40 kg/m^2^ completed the EES; 81 patients with BMI < 40 kg/m^2^ and 193 patients with BMI ≥ 40 kg/m^2^ completed the BIS-11; 69 patients with BMI < 40 kg/m^2^ and 153 patients with BMI ≥ 40 kg/m^2^ completed the TEMPS-A. Statistical analysis comparing the two groups revealed counterintuitive and interesting findings (Table 4). In fact, results showed that patients with severe obesity (BMI ≥ 40 kg/m^2^) present significantly lower scores on the EES (*p* = 0.017) and higher scores on the hyperthymic temperament subscale (*p* = 0.050) compared to the group of patients without severe obesity (BMI < 40 kg/m^2^). The sex ratio between the two groups is provided in the Supplementary Materials (Table S3).

## 4. Discussion

To the best of our knowledge, this represents the first study aimed at investigating the relationship between impulsivity dimensions and affective temperaments with respect to disordered eating behavior, namely emotional eating, in a sample of obese patients seeking bariatric surgery.

In line with most previous studies on this topic [24,45,46], a predominance of women was detected in our sample, constituting almost three-quarters of the participants. The average age was 47 years, and the vast majority of participants were classified as obese class III, indicative of severe obesity. Furthermore, the educational level was generally lower than that of the general Italian population, as also highlighted by previous evidence on similar samples [24]. Moreover, we found how emotional eaters accounted for approximately 40% in our sample, in line with preexisting evidence for clinic samples of bariatric surgery candidates [47,48]. Consistent with the growing evidence of a particularly high rate of psychiatric comorbidities in obese patients referred for bariatric surgery, in the current sample, more than one-third of participants were diagnosed with at least one lifetime psychiatric disorder. Interestingly, we observed that both familiarity for psychiatric disorders and psychiatric diagnoses in comorbidity, whether lifetime or current, were significantly higher in the subgroup of patients with emotional eating than in those without. This represents a finding of particular interest as it suggests that in patients with severe obesity and emotional eating there may be bridging clinical features with psychiatric disorders, which may represent a common psychopathological matrix. Specifically, we observed a significantly higher lifetime presence of anxiety disorders and eating disorders in our sample. This finding enhances current evidence, which indicates that anxiety disorders represent the most frequent comorbidity in obese patients who are candidates for bariatric surgery [49], along with mood disorders [50,51], and eating disorders [52]. Indeed, significantly higher rates of lifetime anxiety disorders were highlighted in participants with EE from our sample, both for generalized anxiety disorder (GAD) and panic disorder (PD). This result confirms our expectations, as it is nowadays widely known in scientific literature how food and eating can be used to cope with negative emotional dysregulation [53]. Indeed anxiety symptoms and disorders frequently co-occur with overeating and emotional eating [54]. As mentioned, increasing evidence has also highlighted substantially higher prevalence of EDs in bariatric patients, although considerable variability from study to study is reported, ranging approximately from 0.6–39% for Bulimia Nervosa (BN) and 5–49% for binge-eating disorders (BED) [24,25,52,55,56,57]. In our sample as well, a significantly higher prevalence of overall lifetime EDs was observed in participants with EE than in the comparison group, with rates of 3% for BN and 8% for BED. Given the higher prevalence of psychiatric disorders in participants with EE, our study also revealed a correspondingly higher current intake of psychotropic drugs in such patients. This result must be taken into account, as many psychotropic drugs can be an iatrogenic cause of weight gain, with a significant metabolic impact of increased body fat mass. In fact, a recent meta-analysis showed that lifestyle interventions are statistically insignificant in reducing psychotropic drug-induced weight gain among people with severe mental illness [58]. This is an important factor to consider in obese patients, especially in the period following surgery, as lifestyle or psychoeducational interventions may not be sufficient to prevent weight regain in patients undergoing psychiatric treatment. For this reason, it would be desirable to switch to psychotropic drugs with less impact on the metabolic profile.

When investigating the common psychopathological interplay between severe obesity with DEBs, and psychiatric disorders and features, we cannot refrain from considering the biological grounds of such occurrences. Indeed, both clinical and brain imaging research suggest how obesity, EDs, and DEBs share a common neural model across a spectrum of reflective versus impulsive eating behaviors [14,59,60]. Indeed, most neurobiological models propose an imbalance between “top-down” cognitive control-related circuitry in the pre-frontal cortex (PFC) and the “bottom-up” interoceptive and reward-processing brain areas. Specifically, a deficiency in top-down inhibitory control over salient stimuli (such as high palatable food) have been described in PFC regions such as the dorsolateral prefrontal cortex (DLPFC), the medial prefrontal cortex (mPFC), the orbitofrontal cortex (OFC), and the anterior cingulate cortex (ACC), which are strictly interlinked to ensure complex tasks such as cognitive evaluation and executive functioning [61,62,63]. Subsequently, greater activation in “bottom-up” reward and salience networks has been detected, mediated by the anterior insula, the ventral striatum (nucleus accumbens), and the amygdala [64]. Thus, behaviors relating to weight gain, such as the emotional eating, grazing, and overconsumption of palatable foods, are the result of bottom-up impulses (i.e., the impulsive system) that are not sufficiently regulated via top-down cognitive control processes (i.e., the reflective system) [65,66,67]. This has been associated with an increased drive for cravings mediated by the ventral limbic network, which could lead to food addiction and loss of control over eating, with recurrence of binge-eating. The impulsiveness dimension therefore plays a crucial role in this process; indeed activity in brain regions critical for executive function is also associated with impulsivity [68,69]. Our study corroborates this dual model, as a positive and statistically significant correlation between emotional eating and impulsivity emerged in our sample of obese patients seeking bariatric surgery. Notably, the total score of the EES scale on emotional eating positively correlates not only with the total score of the BIS scale on impulsivity but also with both of its sub-dimensions, namely “attentional impulsivity” and “motor impulsivity”. Thus, “attentional impulsivity” might result from a lack of cognitive inhibitory control over salient stimuli, while the “motor impulsivity” reflects a reduced ability to suppress an automatic behavior driven by the reward system. Then, impulsivity contributes to the development of DEBs and excessive weight gain seen in extreme obesity and may impact the results of bariatric surgery, representing a negative prognostic factor [70,71,72,73]. This is especially relevant when considering the close interconnection between the impulsivity dimension and the enduring characteristic traits of some affective temperaments. In fact, previous evidence has highlighted a positive correlation between cyclothymic temperaments in developing DEBs and multiple weight cycling (MWC) in obese patients, notwithstanding the absence of a formal psychiatric diagnosis, whereas the hyperthymic temperament showed a protective effect on binge-eating and MWC [74]. Cyclothymic temperament is characterized by affective reactivity, combined with interpersonal sensitivity, limited self-awareness, impulsivity, and a tendency toward sensation-seeking and self-stimulatory behaviors [31,75]. Given that DEBs, such as emotional eating and binge-eating, represent the result of a dysfunctional regulatory strategy to impulsively cope with negative emotions, it should come as no surprise that cyclothymic, anxious, and irritable temperaments may represent a constitutional predisposition that may facilitate and maintain over time both the emergence of maladaptive behaviors (e.g., emotional-eating, binge-eating, binge-purging, snaking, grazing) and as the development of obesity. As a matter of fact, in our study sample, the total emotional eating score correlates positively and statistically significantly with the cyclothymic, depressive, irritable, and anxious temperament, but not with the hyperthymic temperament. A further interesting finding emerged when dividing our sample based on the severity of the patients’ obesity. Patients with higher BMI (BMI ≥ 40, Class III obesity) had lower scores on the EES scale and higher scores on the hyperthymic temperament subscale. This apparently counterintuitive finding might suggest that the most severely obese patients tend to “mask” the severity of their eating disorder (low scores on the EES) and their temperament (high scores on the hyperthymic subscale), trying to mask their psychopathological symptoms as much as possible in the self-report assessment instruments to obtain approval for bariatric surgery.

When discussing our results, some limitations must be considered. Firstly, the cross-sectional study design, which did not allow us to draw any considerations regarding trends over time, making it challenging to determine the causal relationships between variables. Secondly, evaluations were conducted in the framework of bariatric surgery assessments with self-reported questionnaires, meaning that patients may have underreported psychiatric symptoms to get approval for surgery. Finally, although no patients in our sample were currently on GLP-1 agonists therapy at the moment of the assessment, this must be carefully evaluated during both pre- and post-surgical periods, as these medications have been shown to impact hunger/satiety interoceptive signals and may potentially influence the scores on some scales such as the EES [76,77,78].

## 5. Conclusions

Despite its limitations, our research underscores a substantial link between emotional eating in individuals suffering from severe obesity and specific temperamental traits, including cyclothymic, irritable, anxious, and depressive temperaments, as well as the impulsivity dimension. This relationship suggests that problematic eating behaviors and underlying temperamental characteristics are intricately connected, potentially exerting a bidirectional psychopathological influence on obese individuals. These findings highlight the critical importance of thoroughly assessing such temperamental and behavioral factors in patients who are candidates for bariatric surgery. Given the complexity of these interactions, a comprehensive evaluation can better inform treatment plans and improve patient outcomes. Moreover, further research using longitudinal designs is needed to assess the long-term impact of these factors. These studies should include follow-up assessments conducted after bariatric surgery and investigate the influence of GLP-1 agonist medications to gain deeper insights into the long-term impact of these variables on the outcomes of surgery. Such research will be useful in advancing our understanding of the interplay between psychological and behavioral variables in the treatment of severe obesity and should be taken into account when delivering a personalized long-term therapeutic approach.

## Figures and Tables

**Table 1:** Sociodemographic characteristics of the sample (N = 304).

**Female gender** (*n*, %)	217 (71.4%)
**Age, years** (mean, sd)	47.49 (10.93)
**BMI, kg/m^2^** (mean, sd)	43.27 (6.27)
**Marital status** (*n*, %)	
Single	45 (14.8%)
Married	209 (68.7%)
Divorced	40 (13.2%)
Widowed	10 (3.3%)
**Education** (*n*, %)	
Degree	35 (11.5%)
High school diploma	125 (41.1%)
Secondary/elementary school diploma	144 (47.4%)
**Work status** (*n*, %)	
Unemployed	63 (20.7%)
Employed	241 (79.3%)

**Abbreviations**: BMI, Body Mass Index.

**Table 2:** Correlation of the Emotional Eating Scale (EES) total score with the subscales and total score of the Barratt Impulsiveness Scale (BIS-11) and the Temperament Evaluation of the Memphis, Pisa, Paris, and San Diego Autoquestionnaire (TEMPS-A).

	BIS-11 Attentive Impulsivity	BIS-11 Motor Impulsivity	BIS-11 Non-Planning Impulsivity	BIS-11 Total Score	TEMPS-A Cyclothymic Temperament	TEMPS-A Depressive Temperament	TEMPS-A Irritable Temperament	TEMPS-A Hyperthymic Temperament	TEMPS-A Anxious Temperament
Pearson’s correlation	0.299	0.142	0.059	0.187	0.372	0.274	0.178	0.114	0.166
Significance (*p*)	<0.001	0.024	0.347	0.003	<0.001	<0.001	0.013	0.116	0.020
Sample Size (N)	254	254	254	254	198	198	195	193	196

**Table 3:** Comparisons of clinical variables between patients with and without emotional eating (EE).

	No-EE (N = 159, 61%)	EE (N = 103, 39%)	χ^2^/T	OR (95% C.I.)	*p*
Age (mean, sd)	47.84 (11.17)	45.45 (10.76)	1.720	-	0.087
Female gender (*n*, %)	107 (67.3%)	80 (77.7%)	3.293	1.69 (0.96–2.99)	0.070
BMI (mean, sd)	43.70 (6.45)	42.69 (6.30)	1.242	-	0.215
Lifetime psychiatric comorbidities (*n*, %)					
Bipolar Disorder	10 (6.3%)	10 (9.7%)	1.037	1.60 (0.64–4.00)	0.309
Major Depressive Disorder	11 (6.9%)	11 (10.7%)	1.150	1.61 (0.67–3.86)	0.284
Anxiety Disorders	11 (6.9%)	18 (17.5%)	7.078	2.85 (1.28–6.32)	0.008
Eating Disorders	7 (4.4%)	13 (12.6%)	5.988	3.14 (1.21–8.15)	0.014
Any psychiatric disorder	45 (28.3%)	43 (41.7%)	5.066	1.82 (1.08–3.06)	0.024
Psychiatric family history (*n*, %)					
Major Depressive Disorder	19 (11.9%)	15 (14.6%)	0.378	1.26 (0.61–2.60)	0.539
Bipolar Disorder	12 (7.5%)	14 (13.6%)	2.555	1.93 (0.85–4.35)	0.110
Anxiety Disorder	7 (4.4%)	19 (18.4%)	13.792	4.91 (1.98–12.16)	<0.001
Eating Disorders	2 (1.3%)	1 (1.0%)	0.045	0.77 (0.07–8.60)	0.831
Suicidality	3 (1.9%)	7 (6.8%)	4.104	3.79 (0.96–15.01)	0.043
Any psychiatric disorder	34 (21.4%)	34 (33.0%)	4.396	1.81 (1.04–3.17)	0.036
Current psychopharmacologic treatment					
Mood stabilizers	4 (2.5%)	10 (9.8%)	6.502	4.21 (1.29–13.82)	0.011
Antidepressants	14 (5.3%)	18 (6.9%)	4.383	2.19 (1.04–4.64)	0.036
Antipsychotics	0 (0.0%)	2 (0.8%)	3.111	-	0.078

**Table 4:** Comparisons of Emotional Eating Scale (EES), Barratt Impulsiveness Scale (BIS-11), and Temperament Evaluation of the Memphis, Pisa, Paris, and San Diego Autoquestionnaire (TEMPS-A) between patients with BMI < 40 kg/m^2^ and patients with BMI ≥ 40 kg/m^2^.

	BMI < 40 kg/m^2^	BMI ≥ 40 kg/m^2^	Z	*p*
EES total score, mean (sd)	26.53 (21.76)	20.62 (20.7)	−2.385	0.017
BIS-11 Attentive impulsivity	12.85 (2.67)	13.07 (3.49)	−0.018	0–985
BIS-11 Motor impulsivity	19.21 (3.47)	19.31 (4.51)	−0.438	0.661
BIS-11 Non-planning impulsivity	24.86 (5.11)	24.54 (5.23)	−0.373	0.709
BIS-11 Total score	56.93 (8.46)	56.92 (10.63)	−0.384	0.701
TEMPS-A cyclothymic temperament	2.66 (3.13)	2.35 (2.57)	−0.139	0.890
TEMPS-A depressive temperament	0.98 (1.52)	1.24 (1.62)	−1.748	0.080
TEMPS-A irritable temperament	0.98 (1.31)	0.87 (1.25)	−0.735	0.463
TEMPS-A hyperthymic temperament	5.09 (2.50)	5.92 (2.40)	−1.949	0.050
TEMPS-A anxious temperament	1.29 (1.12)	1.24 (1.05)	−0.217	0.786

## Data Availability

The data presented in this study are available on request from the corresponding author due to privacy reasons.

## Supplementary Materials

The following supporting information can be downloaded at: https://www.mdpi.com/article/10.3390/brainsci15040372/s1, Table S1: Correlation of the Emotional Eating Scale (EES) total score with the subscales and total score of the Barratt Impulsiveness Scale (BIS-11) and the Temperament Evaluation of the Memphis, Pisa, Paris, and San Diego Autoquestionnaire (TEMPS-A) including only female patients; Table S2: Correlation of the Emotional Eating Scale (EES) total score with the subscales and total score of the Barratt Impulsiveness Scale (BIS-11) and the Temperament Evaluation of the Memphis, Pisa, Paris, and San Diego Autoquestionnaire (TEMPS-A) including only male patients; Table S3: Sex ratio in the BMI< and >40 kg/m^2^ groups of patients.

## References

[1] Chooi Y.C., Ding C., Magkos F. (2019). The epidemiology of obesity. Metabolism.

[2] Must A., Spadano J., Coakley E.H., Field A.E., Colditz G., Dietz W.H. (1999). The disease burden associated with overweight and obesity. JAMA.

[3] Gravina D., Keeler J.L., Akkese M.N., Bektas S., Fina P., Tweed C., Willmund G.D., Treasure J., Himmerich H. (2023). Randomized Controlled Trials to Treat Obesity in Military Populations: A Systematic Review and Meta-Analysis. Nutrients.

[4] Meldrum D.R., Morris M.A., Gambone J.C. (2017). Obesity pandemic: Causes, consequences, and solutions-but do we have the will?. Fertil. Steril..

[5] Heymsfield S.B., Wadden T.A. (2017). Mechanisms, Pathophysiology, and Management of Obesity. N. Engl. J. Med..

[6] Dakanalis A., Mentzelou M., Papadopoulou S.K., Papandreou D., Spanoudaki M., Vasios G.K., Pavlidou E., Mantzorou M., Giaginis C. (2023). The Association of Emotional Eating with Overweight/Obesity, Depression, Anxiety/Stress, and Dietary Patterns: A Review of the Current Clinical Evidence. Nutrients.

[7] Nagata J.M., Garber A.K., Tabler J.L., Murray S.B., Bibbins-Domingo K. (2018). Prevalence and Correlates of Disordered Eating Behaviors Among Young Adults with Overweight or Obesity. J. Gen. Intern. Med..

[8] van Strien T., Cebolla A., Etchemendy E., Gutiérrez-Maldonado J., Ferrer-García M., Botella C., Baños R. (2013). Emotional eating and food intake after sadness and joy. Appetite.

[9] Konttinen H., Männistö S., Sarlio-Lähteenkorva S., Silventoinen K., Haukkala A. (2010). Emotional eating, depressive symptoms and self-reported food consumption. A population-based study. Appetite.

[10] Adam T.C., Epel E.S. (2007). Stress, eating and the reward system. Physiol. Behav..

[11] Carmassi C., Bertelloni C.A., Massimetti G., Miniati M., Stratta P., Rossi A., Dell L. (2015). Impact of DSM-5 PTSD and gender on impaired eating behaviors in 512 Italian earthquake survivors. Psychiatry Res..

[12] Iceta S., Rodrigue C., Legendre M., Daoust J., Flaudias V., Michaud A., Bégin C. (2021). Cognitive function in binge eating disorder and food addiction: A systematic review and three-level meta-analysis. Prog. Neuropsychopharmacol. Biol. Psychiatry.

[13] McIntyre R.S., McElroy S.L., Konarski J.Z., Soczynska J.K., Bottas A., Castel S., Wilkins K., Kennedy S.H. (2007). Substance use disorders and overweight/obesity in bipolar I disorder: Preliminary evidence for competing addictions. J. Clin. Psychiatry.

[14] Dassen F.C.M., Houben K., Allom V., Jansen A. (2018). Self-regulation and obesity: The role of executive function and delay discounting in the prediction of weight loss. J. Behav. Med..

[15] Bénard M., Camilleri G.M., Etilé F., Méjean C., Bellisle F., Reach G., Hercberg S., Péneau S. (2017). Association between Impulsivity and Weight Status in a General Population. Nutrients.

[16] Ouellette A.S., Rodrigue C., Lemieux S., Tchernof A., Biertho L., Bégin C. (2017). An examination of the mechanisms and personality traits underlying food addiction among individuals with severe obesity awaiting bariatric surgery. Eat. Weight. Disord..

[17] Miller-Matero L.R., Bryce K., Saulino C.K., Dykhuis K.E., Genaw J., Carlin A.M. (2018). Problematic Eating Behaviors Predict Outcomes After Bariatric Surgery. Obes. Surg..

[18] Bonder R., Davis C., Kuk J.L., Loxton N.J. (2018). Compulsive “grazing” and addictive tendencies towards food. Eur. Eat. Disord. Rev..

[19] Goodpaster K.P.S., Marek R.J., Lavery M.E., Ashton K., Merrell Rish J., Heinberg L.J. (2016). Graze eating among bariatric surgery candidates: Prevalence and psychosocial correlates. Surg. Obes. Relat. Dis..

[20] Opolski M., Chur-Hansen A., Wittert G. (2015). The eating-related behaviours, disorders and expectations of candidates for bariatric surgery. Clin. Obes..

[21] Frayn M., Knäuper B. (2018). Emotional eating and weight in adults: A review. Curr. Psychol..

[22] Amianto F., Lavagnino L., Abbate-Daga G., Fassino S. (2011). The forgotten psychosocial dimension of the obesity epidemic. Lancet.

[23] Duarte-Guerra L.S., Coêlho B.M., Santo M.A., Lotufo-Neto F., Wang Y.P. (2017). Morbidity persistence and comorbidity of mood, anxiety, and eating disorders among preoperative bariatric patients. Psychiatry Res..

[24] Barbuti M., Brancati G.E., Calderone A., Fierabracci P., Salvetti G., Weiss F., Carignani G., Santini F., Perugi G. (2022). Prevalence of mood, panic and eating disorders in obese patients referred to bariatric surgery: Patterns of comorbidity and relationship with body mass index. Eat. Weight. Disord..

[25] Dawes A.J., Maggard-Gibbons M., Maher A.R., Booth M.J., Miake-Lye I., Beroes J.M., Shekelle P.G. (2016). Mental Health Conditions Among Patients Seeking and Undergoing Bariatric Surgery: A Meta-analysis. JAMA.

[26] McElroy S.L., Kotwal R., Malhotra S., Nelson E.B., Keck P.E., Nemeroff C.B. (2004). Are mood disorders and obesity related? A review for the mental health professional. J. Clin. Psychiatry.

[27] Carmassi C., Musetti L., Cambiali E., Violi M., Simoncini M., Fantasia S., Massoni L., Massimetti G., Nannipieri M., Dell’Osso L. (2025). Exploring the relationship between problematic eating behaviors and bipolar disorder: A study on candidates for bariatric surgery. J. Affect. Disord..

[28] Akiskal K.K., Akiskal H.S. (2005). The theoretical underpinnings of affective temperaments: Implications for evolutionary foundations of bipolar disorder and human nature. J. Affect. Disord..

[29] Cassano G.B., Akiskal H.S., Perugi G., Musetti L., Savino M. (1992). The importance of measures of affective temperaments in genetic studies of mood disorders. J. Psychiatr. Res..

[30] Akiskal H.S., Akiskal K.K., Haykal R.F., Manning J.S., Connor P.D. (2005). TEMPS-A: Progress towards validation of a self-rated clinical version of the Temperament Evaluation of the Memphis, Pisa, Paris, and San Diego Autoquestionnaire. J. Affect. Disord..

[31] Perugi G., Toni C., Maremmani I., Tusini G., Ramacciotti S., Madia A., Fornaro M., Akiskal H.S. (2012). The influence of affective temperaments and psychopathological traits on the definition of bipolar disorder subtypes: A study on bipolar I Italian national sample. J. Affect. Disord..

[32] Ramacciotti C.E., Paoli R.A., Ciapparelli A., Marcacci G., Placidi G.E., Dell’Osso L., Garfinkel P.E. (2004). Affective temperament in the eating disorders. Eat. Weight. Disord..

[33] Amann B., Mergl R., Torrent C., Perugi G., Padberg F., El-Gjamal N., Laakmann G. (2009). Abnormal temperament in patients with morbid obesity seeking surgical treatment. J. Affect. Disord..

[34] Lesiewska N., Borkowska A., Junik R., Kamińska A., Pulkowska-Ulfig J., Tretyn A., Bieliński M. (2019). The Association Between Affective Temperament Traits and Dopamine Genes in Obese Population. Int. J. Mol. Sci..

[35] NIH conference (1991). Gastrointestinal surgery for severe obesity. Consensus Development Conference Panel. Ann. Intern. Med..

[36] Flum D.R., Belle S.H., King W.C., Wahed A.S., Berk P., Chapman W., Pories W., Courcoulas A., McCloskey C., Mitchell J. (2009). Perioperative safety in the longitudinal assessment of bariatric surgery. N. Engl. J. Med..

[37] Snyder A.G. (2009). Psychological assessment of the patient undergoing bariatric surgery. Ochsner J..

[38] First M.B., Williams J.B., Karg R.S., Spitzer R.L. (2016). SCID-5-CV: Structured Clinical Interview for DSM-5 Disorders: Clinician Version.

[39] Arnow B., Kenardy J., Agras W.S. (1995). The Emotional Eating Scale: The development of a measure to assess coping with negative affect by eating. Int. J. Eat. Disord..

[40] Patton J.H., Stanford M.S., Barratt E.S. (1995). Factor structure of the Barratt impulsiveness scale. J. Clin. Psychol..

[41] Preti A., Vellante M., Zucca G., Tondo L., Akiskal K., Akiskal H. (2010). The Italian version of the validated short TEMPS-A: The temperament evaluation of Memphis, Pisa, Paris and San Diego. J. Affect. Disord..

[42] Akiskal H.S., Mendlowicz M.V., Jean-Louis G., Rapaport M.H., Kelsoe J.R., Gillin J.C., Smith T.L. (2005). TEMPS-A: Validation of a short version of a self-rated instrument designed to measure variations in temperament. J. Affect. Disord..

[43] Elias L.R., Köhler C.A., Stubbs B., Maciel B.R., Cavalcante L.M., Vale A.M.O., Gonda X., Quevedo J., Hyphantis T.N., Soares J.C. (2017). Measuring affective temperaments: A systematic review of validation studies of the Temperament Evaluation in Memphis Pisa and San Diego (TEMPS) instruments. J. Affect. Disord..

[44] Purnell J.Q. (2023). What is Obesity?: Definition as a Disease, with Implications for Care. Gastroenterol. Clin. N. Am..

[45] Lin H.Y., Huang C.K., Tai C.M., Lin H.Y., Kao Y.H., Tsai C.C., Hsuan C.F., Lee S.L., Chi S.C., Yen Y.C. (2013). Psychiatric disorders of patients seeking obesity treatment. BMC Psychiatry.

[46] Flum D.R., Dellinger E.P. (2004). Impact of gastric bypass operation on survival: A population-based analysis. J. Am. Coll. Surg..

[47] Walfish S. (2004). Self-assessed emotional factors contributing to increased weight gain in pre-surgical bariatric patients. Obes. Surg..

[48] Guerdjikova A.I., West-Smith L., McElroy S.L., Sonnanstine T., Stanford K., Keck P.E. (2007). Emotional eating and emotional eating alternatives in subjects undergoing bariatric surgery. Obes. Surg..

[49] Duarte-Guerra L.S., Coêlho B.M., Santo M.A., Wang Y.P. (2015). Psychiatric disorders among obese patients seeking bariatric surgery: Results of structured clinical interviews. Obes. Surg..

[50] Barbuti M., Carignani G., Weiss F., Calderone A., Santini F., Perugi G. (2021). Mood disorders comorbidity in obese bariatric patients: The role of the emotional dysregulation. J. Affect. Disord..

[51] Zhao Z., Okusaga O.O., Quevedo J., Soares J.C., Teixeira A.L. (2016). The potential association between obesity and bipolar disorder: A meta-analysis. J. Affect. Disord..

[52] Dahl J.K., Eriksen L., Vedul-Kjelsås E., Strømmen M., Kulseng B., Mårvik R., Holen A. (2010). Prevalence of all relevant eating disorders in patients waiting for bariatric surgery: A comparison between patients with and without eating disorders. Eat. Weight. Disord..

[53] Barbuti M., Carignani G., Weiss F., Calderone A., Fierabracci P., Salvetti G., Menculini G., Tortorella A., Santini F., Perugi G. (2023). Eating disorders and emotional dysregulation are associated with insufficient weight loss after bariatric surgery: A 1-year observational follow-up study. Eat. Weight. Disord..

[54] Fonseca N., Costa M.A., Gosmann N.P., Dalle Molle R., Gonçalves F.G., Silva A.C., Rodrigues Y., Silveira P.P., Manfro G.G. (2023). Emotional eating in women with generalized anxiety disorder. Trends Psychiatry Psychother..

[55] Sekuła M., Boniecka I., Paśnik K. (2019). Bulimia nervosa in obese patients qualified for bariatric surgery–clinical picture, background and treatment. Videosurgery Other Miniinvasive Tech..

[56] Morseth M.S., Hanvold S.E., Rø Ø., Risstad H., Mala T., Benth J., Engström M., Olbers T., Henjum S. (2016). Self-Reported Eating Disorder Symptoms Before and After Gastric Bypass and Duodenal Switch for Super Obesity--a 5-Year Follow-Up Study. Obes. Surg..

[57] Rosenberger P.H., Henderson K.E., Grilo C.M. (2006). Psychiatric disorder comorbidity and association with eating disorders in bariatric surgery patients: A cross-sectional study using structured interview-based diagnosis. J. Clin. Psychiatry.

[58] Mohanty K., Gandhi S., Krishna Prasad M., John A.P., Bhaskarapillai B., Malo P., Thirthalli J. (2024). Effectiveness of lifestyle intervention on prevention/management of antipsychotic-induced weight gain among persons with severe mental illness: A systematic review and meta-analysis. J. Health Psychol..

[59] Brooks S.J., Rask-Andersen M., Benedict C., Schiöth H.B. (2012). A debate on current eating disorder diagnoses in light of neurobiological findings: Is it time for a spectrum model?. BMC Psychiatry.

[60] Marsh R., Steinglass J.E., Gerber A.J., Graziano O’Leary K., Wang Z., Murphy D., Walsh B.T., Peterson B.S. (2009). Deficient activity in the neural systems that mediate self-regulatory control in bulimia nervosa. Arch. Gen. Psychiatry.

[61] Brooks S.J., O’Daly O.G., Uher R., Friederich H.C., Giampietro V., Brammer M., Williams S.C., Schiöth H.B., Treasure J., Campbell I.C. (2011). Differential neural responses to food images in women with bulimia versus anorexia nervosa. PLoS ONE.

[62] Bronleigh M., Baumann O., Stapleton P. (2022). Neural correlates associated with processing food stimuli in anorexia nervosa and bulimia nervosa: An activation likelihood estimation meta-analysis of fMRI studies. Eat. Weight. Disord..

[63] Kaye W.H., Wagner A., Fudge J.L., Paulus M. (2011). Neurocircuity of eating disorders. Curr. Top. Behav. Neurosci..

[64] Ravichandran S., Bhatt R.R., Pandit B., Osadchiy V., Alaverdyan A., Vora P., Stains J., Naliboff B., Mayer E.A., Gupta A. (2021). Alterations in reward network functional connectivity are associated with increased food addiction in obese individuals. Sci. Rep..

[65] Hofmann W., Friese M., Strack F. (2009). Impulse and Self-Control From a Dual-Systems Perspective. Perspect. Psychol. Sci..

[66] Meule A., Hofmann J., Weghuber D., Blechert J. (2016). Impulsivity, perceived self-regulatory success in dieting, and body mass in children and adolescents: A moderated mediation model. Appetite.

[67] Strack F., Deutsch R. (2004). Reflective and impulsive determinants of social behavior. Pers. Soc. Psychol. Rev..

[68] Raman J., Smith E., Hay P. (2013). The clinical obesity maintenance model: An integration of psychological constructs including mood, emotional regulation, disordered overeating, habitual cluster behaviours, health literacy and cognitive function. J. Obes..

[69] Day J., Ternouth A., Collier D.A. (2009). Eating disorders and obesity: Two sides of the same coin?. Epidemiol. Psichiatr. Soc..

[70] Geerts M.M., van den Berg E.M., van Riel L., Peen J., Goudriaan A.E., Dekker J.J.M. (2021). Behavioral and psychological factors associated with suboptimal weight loss in post-bariatric surgery patients. Eat. Weight. Disord..

[71] Yeo D., Toh A., Yeo C., Low G., Yeo J.Z., Aung M.O., Rao J., Kaushal S. (2021). The impact of impulsivity on weight loss after bariatric surgery: A systematic review. Eat. Weight. Disord..

[72] Schag K., Mack I., Giel K.E., Ölschläger S., Skoda E.M., von Feilitzsch M., Zipfel S., Teufel M. (2016). The Impact of Impulsivity on Weight Loss Four Years after Bariatric Surgery. Nutrients.

[73] Sarwer D.B., Allison K.C., Wadden T.A., Ashare R., Spitzer J.C., McCuen-Wurst C., LaGrotte C., Williams N.N., Edwards M., Tewksbury C. (2019). Psychopathology, disordered eating, and impulsivity as predictors of outcomes of bariatric surgery. Surg. Obes. Relat. Dis..

[74] Scumaci E., Marzola E., Abbate-Daga G., Pellegrini M., Ponzo V., Goitre I., Benso A., Broglio F., Belcastro S., Crespi C. (2021). Affective temperaments and obesity: Is there an association with binge eating episodes and multiple weight cycling?. J. Affect. Disord..

[75] Perugi G., Toni C., Passino M.C., Akiskal K.K., Kaprinis S., Akiskal H.S. (2006). Bulimia nervosa in atypical depression: The mediating role of cyclothymic temperament. J. Affect. Disord..

[76] Nicolau J., Pujol A., Tofé S., Bonet A., Gil A. (2022). Short term effects of semaglutide on emotional eating and other abnormal eating patterns among subjects living with obesity. Physiol. Behav..

[77] Wong L., Stammers L., Churilov L., Price S., Ekinci E., Sumithran P. (2020). Emotional eating in patients attending a specialist obesity treatment service. Appetite.

[78] van Ruiten C.C., Ten Kulve J.S., van Bloemendaal L., Nieuwdorp M., Veltman D.J., RG I.J. (2022). Eating behavior modulates the sensitivity to the central effects of GLP-1 receptor agonist treatment: A secondary analysis of a randomized trial. Psychoneuroendocrinology.

