# Supplementary material for: Emotional Eating, Impulsivity, and Affective Temperaments in a Sample of Obese Candidates for Bariatric Surgery: Which Linkage?
Source: Brain Sci. 2025 Apr 3;15(4):372. doi: 10.3390/brainsci15040372 (PMC12025757; doi:10.3390/brainsci15040372)
Supplement: Supplementary file 1 [file brainsci-15-00372-s001.zip › brainsci-3515730-supplementary.pdf]

Supplementary Materials

Table S1. Correlation of the Emotional Eating Scale (EES) total score with the subscales and total score of the Barratt Impulsiveness Scale (BIS-11) and the Temperament Evaluation of the Memphis, Pisa, Paris, and San Diego Autoquestionnaire (TEMPS-A) including only female patients.

	BIS-11 Attentive impulsivity	BIS-11 Motor impulsivity	BIS-11 Non-planning impulsivity	BIS-11 total score	TEMPS-A Cyclothymic temperament	TEMPS-A Depressive temperament	TEMPS-A Irritable temperament	TEMPS-A Hyperthymic temperament	TEMPS-A Anxious temperament
Pearson's correlation	0.291	0.130	0.004	0.154	0.361	0.338	0.241	0.186	0.193
Significance (*p*)	**<0.001**	0.082	0.953	**0.039**	**<0.001**	**<0.001**	**0.005**	**0.032**	**0.024**
Sample Size (N)	180	180	180	180	139	139	136	134	137

Table S2. Correlation of the Emotional Eating Scale (EES) total score with the subscales and total score of the Barratt Impulsiveness Scale (BIS-11) and the Temperament Evaluation of the Memphis, Pisa, Paris, and San Diego Autoquestionnaire (TEMPS-A) including only male patients.

	BIS-11 Attentive impulsivity	BIS-11 Motor impulsivity	BIS-11 Non-planning impulsivity	BIS-11 total score	TEMPS-A Cyclothymic temperament	TEMPS-A Depressive temperament	TEMPS-A Irritable temperament	TEMPS-A Hyperthymic temperament	TEMPS-A Anxious temperament
Pearson's correlation	0.294	0.135	0.169	0.230	0.383	0.119	0.068	0.071	0.121
Significance (*p*)	**0.011**	0.250	0.149	**0.049**	**0.003**	0.369	0.610	0.594	0.361
Sample Size (N)	74	74	74	74	59	59	59	59	59

Table S3. Sex ratio in the BMI< and >40 kg/m² groups of patients.

	**BMI < 40 kg/m²**	**BMI > 40 kg/m²**	**OR (95% C.I.)**	**p**
Female gender (n, %)	72 (79.1%)	144 (67.9%)	0.56 (0.31-1.00)	0.048
